# Multicenter Retrospective Analysis of 702 Pediatric Cases of Bone and Joint Infections: Definition of Clinical and Biological Features to Discriminate *K. kingae*, *S. aureus*, and Other Bacterial Infections

**DOI:** 10.3390/pathogens14020147

**Published:** 2025-02-04

**Authors:** Marco Roversi, Francesca Pignatelli, Giacomo De Marco, Oscar Vazquez, Dimitri Ceroni, Antonio Musolino, Marco Cirillo, Laura Lancella, Alberto Villani, Andrzej Krzysztofiak, on behalf of the Osteomyelitis Collaborative Study Group

**Affiliations:** 1 UOC Trials, Bambino Gesù Children’s Hospital, IRCCS, 00165 Rome, Italy; 2 PhD Program in Immunology, Molecular Medicine and Applied Biotechnology, University of Rome Tor Vergata, 00133 Rome, Italy; 3 Infectious Disease Unit, Bambino Gesù Children’s Hospital, IRCCS, 00165 Rome, Italylaura.lancella@opbg.net (L.L.); 4 Pediatric Orthopedic Unit, Pediatric Surgery Service, Geneva University Hospitals, 1206 Geneva, Switzerland; 5 General Pediatrics and ED II Level, Bambino Gesù Children’s Hospital IRCCS, 00165 Rome, Italy; antoniomusolino931@gmail.com (A.M.);; 6 Diagnostic and Interventional Radiology Unit, Bambino Gesù Children’s Hospital, IRCCS, 00165 Rome, Italy; 7 System Medicine Department, Tor Vergata University, 00133 Rome, Italy

**Keywords:** osteoarticular infectious, bone infections, joint infections, osteomyelitis, septic arthritis, pediatric, children, *K. kingae*, *S. aureus*

## Abstract

This study aimed to identify key differences between *K. kingae* infections and those caused by *S. aureus* or other pathogens. Differentiating these infections is crucial due to their nonspecific clinical presentations and overlapping laboratory and radiological findings, particularly when isolates are unavailable. We retrospectively analyzed data from 702 pediatric patients with bone and joint infections from 2010 to 2023 across two hospitals. The most common diagnoses were osteomyelitis (35.3%) and arthritis (29.6%), with fever present in 46.0% of cases. Pathogen identification showed *K. kingae* (40.9%) and *S. aureus* (36.5%) as the most frequent. Patients with *K. kingae* were significantly younger (median age 1.5 years) than those with *S. aureus* (10.4 years) or other pathogens (6.8 years) (*p* < 0.001). Fever was more common in *S. aureus* (64.3%) and other pathogens (57.5%) than in *K. kingae* (26.4%) (*p* < 0.001). CRP levels were lower in *K. kingae* infections (median 1.5 mg/dl) compared to *S. aureus* (6.1 mg/dl) and other pathogens (5.0 mg/dl) (*p* < 0.001). *K. kingae* infections were predominantly treated with penicillin–clavulanate and had shorter treatment durations and lower sequelae rates (2.3%) compared to other pathogens (19.0%). These findings emphasize *K. kingae*’s distinct clinical profile and milder course compared to *S. aureus* and other pathogens.

## 1. Introduction

Pediatric osteoarticular infections (OAIs) encompass acute hematogenous osteomyelitis, septic arthritis, osteoarthritis, and other conditions with overlapping clinical presentations. Even today, these conditions pose significant clinical challenges for physicians, as they can lead to severe morbidity and progress to complications such as growth disorders, joint destruction, and permanent disability if not properly treated [1,2]. Based on our experience, sequelae occur in 13.5% of pediatric bacterial osteomyelitis cases [3]. However, other studies report rates ranging from 7.9% to 29%, with younger children and those with severe presentations showing a higher prevalence [4,5].

Diagnosing osteoarticular infections remains challenging due to their nonspecific clinical, laboratory, and radiological features, as well as the low sensitivity of blood cultures in identifying pathogens. The clinical severity of these infections varies widely, ranging from localized cases with minimal systemic symptoms to multifocal disease associated with septic shock [6]. Clinical and biological characteristics are strongly influenced by children’s ages, the causative pathogens, and factors such as comorbidities, immune status and vaccination history, socio-economic conditions, shifts in patterns of immunomodulating diseases, and the emergence of resistant bacteria [7]. Nonspecific signs like irritability, vomiting, or feeding difficulties are common in neonates and young infants, but none reliably indicates the causative pathogen. Laboratory markers like CRP and ESR are typically elevated. However, neither CRP nor ESR effectively differentiates between pathogens. Blood cultures, while central to microbiological diagnosis, yield pathogens in only about 31.2% of cases [8]. Imaging modalities, particularly MRI, are highly sensitive for detecting early bone and soft tissue involvement, but they cannot determine the causative agent from radiological findings alone [9,10]. Microbiological diagnosis improves significantly when direct samples from bones or abscesses are cultured, achieving yields of up to 87% [6,11]. However, invasive procedures are often avoided when blood cultures suffice, or empirical antibiotic therapy produces excellent outcomes. Advances in nucleic acid amplification techniques, such as PCR, have enhanced pathogen detection, especially when prior antibiotic use reduces culture sensitivity [12].

Identifying the causative pathogen is, therefore, the cornerstone of treatment, as it confirms the diagnosis, allows therapy to be tailored, minimizes the unnecessary use of broad-spectrum antibiotics, accounts for regional variations in pathogen prevalence, and optimizes outcomes [13]. Over the past 20 years, the advent and widespread use of nucleic acid amplification assays (NAAAs) have significantly improved the detection of low levels of bacterial agents in clinical samples, reducing the rate of “culture-negative” OAIs [1,14]. This innovative diagnostic approach has provided compelling evidence that *K. kingae* is now the most common pathogen responsible for primary infections in bones, joints, intervertebral disks, and tendon sheaths, particularly in children aged 6–48 months [15,16,17,18]

Interestingly, *K. kingae* OAIs are often characterized by a mild clinical presentation and a limited biological inflammatory response. As a result, young patients frequently exhibit few, if any, of the typical criteria suggestive of an OAI. In contrast, pyogenic OAIs caused by more aggressive pathogens, such as methicillin-sensitive or methicillin-resistant *S. aureus* (MSSA and MRSA), streptococci, or Gram-negative organisms, are more evident. Children affected by these pathogens usually appear quite ill, presenting with high fever and elevated white blood cell (WBC) counts [19,20]. Finally, while imaging modalities, particularly MRI, are highly sensitive for detecting early bone and soft tissue involvement, they cannot identify the causative agent based on radiological findings alone [9,10].

Given the diagnostic complexities and the heterogeneity of causative agents, this study seeks to clarify the clinical, laboratory, and radiological differences between infections caused by *K. kingae*, *S. aureus*, and other pathogens. A deeper understanding of these distinctions is essential for improving pathogen identification, establishing predictive values to differentiate OAIs caused by these three groups of pathogens, and optimizing targeted treatment strategies.

## 2. Materials and Methods

This multicenter retrospective study included pediatric patients diagnosed with bone and joint infections, specifically osteomyelitis, arthritis, osteoarthritis, and spondylodiscitis, between January 2010 and December 2023. The study involved data from Bambino Gesù Children’s Hospital in Rome, Italy, and Hôpitaux Universitaires de Genève, Switzerland. The criteria established by Morrey [21,22] and Morrissey [23] were used to estimate children’s risk of having a bone or joint infection. Patients under 18 years with confirmed diagnoses, supported by clinical, microbiological, imaging, or histopathological findings, were included. Exclusion criteria for the study included cases of chronic osteomyelitis and infections arising from fractures or surgical procedures. Additional exclusion criteria were applied to minimize information bias from incomplete data analysis and selection bias from the inclusion of patients with presumptive or inconsistent diagnoses. These criteria were as follows: (i) absence of a confirmed bacteriological diagnosis, (ii) unavailability of laboratory data, and (iii) cases where the patient did not ultimately receive antibiotic treatment.

Demographic data, including sex and age in years, were retrospectively reviewed alongside clinical information such as admission diagnosis, fever, and the localization of the infection. Laboratory findings included white blood cell count, CRP (mg/dl), and ESR (mm/h). Diagnostic microbiological assessments comprised throat swabs, blood cultures, biopsy cultures, and quantitative polymerase chain reaction (qPCR), with recorded pathogen identification when available.

Imaging data were gathered from modalities including X-rays, ultrasound, CT, MRI, and scintigraphy. Diagnoses of OAIs were confirmed using imaging techniques (plain radiography, scintigraphy, ultrasonography, CT scanner, magnetic resonance imaging) according to established criteria [24]. Children diagnosed with a musculoskeletal infection were further categorized with the following diagnoses: acute hematogenous osteomyelitis, subacute osteomyelitis, septic arthritis, acute osteomyelitis with concomitant septic arthritis, primary spine infection (spondylodiscitis, vertebral osteomyelitis), septic pyomyositis, septic chondritis and septic tenosynovitis.

Antibiotic regimens were detailed, covering types and routes of administration (IV or oral). The duration of IV, oral, and total antibiotic therapy was recorded. Outcomes assessed included the presence of sequelae (e.g., joint dysfunction, deformities, or chronic infections), length of hospital stay (in days), and relapse rates.

### 2.1. Microbiological Methods

Microbiological Methods Blood cultures have been systematically used for trying to isolate the microorganisms responsible for septic arthritis. This study’s blood culture media were BACTEC 9000 (Becton Dickinson, Sparks, ML, USA) for the period before 2009, and following that, the automated blood culture system BD BACTEC FX (Becton Dickinson, Sparks, USA, Maryland) was used. Joint fluid was sent to the laboratory for Gram staining, cell count, and immediate inoculation onto Columbia blood agar (incubated under anaerobic conditions), CDC anaerobe 5% sheep blood agar (incubated under anaerobic conditions), chocolate agar (incubated in a CO_2_-enriched atmosphere), and brain–heart broth. These media were incubated for 10 days. Two PCR assays were also used for bacterial identification when standard cultures were negative. Initial aliquots (100–200 µL) were stored at 80 C until processing for DNA extraction. A universal, broad-range PCR amplification of the 16S rRNAgene was performed using BAK11w, BAK2, and BAK533r primers (Eurogentec, Seraing, Belgium). This study also used a real-time PCR assay targeting the *K. kingae* gene’s rtx toxin from 2007. The assay is designed to detect two independent gene targets from the *K. kingae* rtx toxin locus, namely rtxA and rtxB [25]. This PCR assay detecting *K. kingae* was used to analyze different biological samples, such as synovial fluid, bone or discal biopsy specimens, or peripheral blood. Since September 2009, we have also been carrying out oropharyngeal swab PCR for children from 6 months to 4 years old. It has been demonstrated that this simple technique for the detection of *K. kingae* rtx toxin genes in the oropharynx provides conflicting evidence, indicating that this microorganism is responsible for OAI, while also suggesting that it is not [26].

### 2.2. Statistical Analysis

Statistical analysis was conducted using the open-source statistical software R-studio version 4.1.2. Continuous variables were expressed as means with standard deviations or medians with interquartile ranges, depending on the distribution, and were compared using Student’s *t*-test or the Mann–Whitney U test. Categorical variables were presented as frequencies and percentages and analyzed using chi-square or Fisher’s exact tests. Multivariate logistic regression identified independent predictors of sequelae, with statistical significance set at *p* < 0.05.

## 3. Results

The characteristics of the study sample are outlined in Table 1.

Based on the inclusion and exclusion criteria, 702 individuals were deemed eligible to participate in the study. The median age of 4.2 years (IQR: 1.4–10.4), and 58.3% of participants were male. The most common diagnoses were osteomyelitis (35.3%) and arthritis (29.6%). The most frequently affected anatomical site was the lower limb (70.2%). Fever was reported in 46.0% of patients, and laboratory findings revealed a median WBC count of 10,730 cells/mm^3^ (IQR: 8,030–13,900), CRP levels of 2.5 mg/dl (IQR: 0.8–7.0), and ESR of 32 mm/h (IQR: 17–49). Microbiological analysis revealed *K. kingae* (40.9%) and *S. aureus* (36.5%) as the most prevalent organisms. MRI was the primary imaging modality (91.9%). Initial intravenous (IV) antibiotic therapy frequently included amoxicillin–clavulanate (54.0%), while oral therapy primarily utilized the same agent (35.8%). The median durations of IV, oral, and total antibiotic therapies were 13, 19, and 31 days, respectively. The median hospital stay was 13 days (IQR: 5–25), and 10.2% of patients experienced sequelae.

The comparison between the three main classes of isolated pathogens is shown in Table 2.

Patients infected with *K. kingae* were significantly younger (median age: 1.5 years, IQR 1.11–2.06) than those with *S. aureus* (10.4 years IQR 6.0–13.0) or other pathogens (6.8 years IQR 1.8–11.9) (*p* < 0.001). There were no significant differences in sex distribution across groups (*p* = 0.272). Osteomyelitis was more frequent in cases of *S. aureus* infection (46.8%) compared to *K. kingae* (28.2%) and other pathogens (32.9%) (*p* < 0.001). Arthritis predominated in *K. kingae* infections (50.6%), while *S. aureus* and other pathogens showed a more varied diagnostic profile, including higher rates of “other” diagnoses (16.4% and 27.8%, respectively). Fever was markedly more common in *S. aureus* (64.3%) and other pathogens (57.5%) than in *K. kingae* (26.4%) (*p* < 0.001). Inflammatory markers such as CRP were lowest in *K. kingae* cases (median 1.5 mg/dl, IQR 0.7–3.2) compared to *S. aureus* (6.1 IQR 1.8–11.5) and other pathogens (5.0 IQR 1.0–13.2) (*p* < 0.001). Similarly, median WBC counts were highest in *K. kingae* infections (12100 cells/mm^3^) and lowest among *S. aureus* infections (9565 cells/mm^3^) (*p* < 0.001). MRI was widely utilized across groups, with the highest use seen for *S. aureus* infections (96.5%) (*p* = 0.011). However, X-rays and ultrasound were more frequently employed for *S. aureus* and other pathogens (*p* < 0.001). Intravenous (IV) and oral antibiotic regimens differed significantly. *K. kingae* infections were predominantly treated with amoxicillin–clavulanate IV (51.1%) and oral (54.0%), whereas *S. aureus* and other pathogens required broader-spectrum agents, such as glycopeptides (29.2% and 26.2%, respectively, *p* < 0.001). The duration of IV therapy was the shortest for *K. kingae* (median 3 days IQR 2–3) compared to *S. aureus* (18 days IQR 8–28) and other pathogens (17 days IQR 7–31) (*p* < 0.001). Similarly, the total antibiotic duration was substantially shorter for *K. kingae* (22 days IQR 21–24) versus *S. aureus* (36 days IQR 30–48) and other pathogens (42 days IQR 30–49) (*p* < 0.001). The length of hospital stay was markedly shorter for *K. kingae* (median 4 days IQR 3–5) compared to *S. aureus* (19 days IQR 10–30) and other pathogens (17 days IQR 8–32) (*p* < 0.001). Sequelae were least common in *K. kingae* infections (2.3%), whereas infections with other pathogens showed the highest rate (19.0%) (*p* < 0.001).

## 4. Discussion

In this study, we presented one of the largest documented cohorts of children with osteomyelitis, providing significant epidemiological and clinical data about these conditions. Our analysis revealed a non-uniform distribution of patients across age groups and genders, with a slight predominance of younger children and males. This highlights that OAIs impact children differently based on their age, an observation supported by previous studies. In an epidemiological study, Juchler et al. found that the median age of children with OAIs was 23 months, and 67.7% of affected children were under 4 years old [16]. Our results further emphasize that the age of the child plays a crucial role in determining the pathogen responsible for the infection. Specifically, children with OAIs caused by *K. kingae* were significantly younger than those with infections caused by *S. aureus* or other pathogens. A similar conclusion was reached in a previous study by Coulin et al., who reported a mean age of 19.9 months for children with OAIs caused by *K. kingae*, compared to a mean age of 9.1 years for those with *S. aureus* infections [15].

Our study also underscores that most pathogens exhibit specific tendencies in the types of infections they primarily cause. *S. aureus* was more commonly associated with osteomyelitis, while K. kingae was more frequently linked to septic arthritis, the most common osteoarticular disease caused by this pathogen. When considering the causal pathogen, we observed that *S. aureus* was most often isolated in tibial and femoral osteomyelitis, whereas *K. kingae* predominated in knee septic arthritis and vertebral infections. These associations are not widely reported in the existing literature. Furthermore, our study provides strong support for the hypothesis that *K. kingae* should now be considered the most frequently isolated pathogen in cases of OAIs [16,17,27,28,29], particularly in children aged 6 to 48 months. In this series, *K. kingae* was identified as the causative pathogen in 40.9% of all OAIs, regardless of the patient’s age. In contrast, *S. aureus*, long regarded as the predominant pathogen responsible for pediatric OAIs, ranked second, highlighting a shift in the role of this pathogen in pediatric osteoarticular infections. This finding aligns with previous studies, which have reported *K. kingae* involvement in up to 48.7% of OAIs [15,16,30,31,32,33,34,35,36,37,38,39,40].

Notably, only 46% of children in our sample had a fever at admission, which is notably lower than the 61.7% reported by Dartnell et al. [22]. This difference can be attributed to the fact that our series includes a majority of *K. kingae* infections, a pathogen that is either minimally represented or absent in some older series. In this context, our study also emphasizes that the clinical presentation of OAIs caused by *K. kingae* differs markedly from those caused by MSSA or other pathogens. It is well recognized that young children with *K. kingae*-related OAIs typically present with less acute symptoms compared to the classic presentation of a severely ill child with fever. In our series, we observed that fever was significantly less common in *K. kingae* OAIs (26.4%) compared to *S. aureus* (64.3%) and other pathogens (57.5%). These findings are consistent with those of Coulin et al., who reported in a previous study that only 24% of children with a *K. kingae* OAI had a temperature >38 °C at admission compared to 63% of children with an MSSA OAI.

Consistent with prior studies [23], CRP and white blood cell [WBC] counts in our cohort were either mildly elevated or normal at diagnosis, highlighting their limited sensitivity for both diagnosing and monitoring osteomyelitis. A key finding of our study is that *K. kingae* OAIs are characterized by a less severe inflammatory response, particularly in terms of CRP levels, compared to those caused by *S. aureus* or other pathogens [41,42]. This observation aligns closely with the findings of Coulin et al. [15], who reported mean CRP values of 24 mg/L for OAIs caused by *K. kingae* compared to 81.6 mg/L for those due to *S. aureus*. Regarding ESR values, our study did not reveal any significant differences between the three groups. This finding is somewhat discordant with the results published by Coulin et al., who observed significant differences in this parameter between *K. kingae* and *S. aureus*-induced OAIs. Surprisingly, the overall mean WBC count was higher in children with a *K. kingae* OAI (12,100 cells/mm^3^) compared to those with an MSSA OAI (10,385 cells/mm^3^) or infections caused by other pathogens (9565 cells/mm^3^). This paradox was previously noted by Coulin et al., who suggested that WBC counts below 17,000 cells/mm^3^ are considered normal in children under 4 years of age.

Most patients underwent radiography and/or MRI to confirm the diagnosis and assess the extent of bone infection. Although ultrasound is not typically recommended for diagnosing pediatric bacterial osteomyelitis, we suggest its use to rule out joint or skin involvement when disease spread to adjacent tissues is suspected. In our study, 29.6% of children were diagnosed with concomitant bone and joint infection.

Regarding treatment, approaches can vary significantly and must be tailored to local bacteriological realities. In our study, *K. kingae* infections were primarily managed with intravenous penicillin–clavulanate, followed by a swift transition to oral antibiotics, while *S. aureus* and other pathogens often necessitated broad-spectrum antibiotics, such as glycopeptides. The use of second-line antibiotics was particularly warranted due to the high prevalence of MRSA infections at one of the two hospitals, as well as the need to address complicated osteomyelitis cases following prior treatment failures. In settings with a high MRSA prevalence, adopting an aggressive empirical antibiotic regimen—including bactericidal agents such as glycopeptides, aminoglycosides, or linezolid—may be advisable. As anticipated, the duration of both intravenous and total therapy was shorter for *K. kingae* infections compared to those caused by *S. aureus* or other pathogens, resulting in reduced hospital stays for *K. kingae* cases. Additionally, follow-up assessments revealed that sequelae were more pronounced in children with *S. aureus* infections.

Our study is subject to several limitations. Its retrospective design increased the risk of missing certain cases due to medical coding errors and contributed to a higher proportion of missing data and patients lost to follow-up. A second limitation is that nucleic acid amplification assays (NAAAs), which have advanced significantly in recent years, were not equally accessible at both centers. As a result, many cases may have been overlooked, potentially increasing the number of “culture-negative” OAIs. Despite these limitations, the descriptive data provided valuable insights into the types and locations of osteomyelitis occurring in conjunction with septic arthritis, as well as the pathogens responsible for these infections.

## 5. Conclusions

Our findings underscore the distinct clinical and therapeutic profiles associated with *K. kingae*, *S. aureus*, and other pathogens. *K. kingae* primarily affects younger children with less severe clinical presentations and shorter treatment durations, whereas *S. aureus* and other pathogens involve older children and are associated with more complex clinical courses and greater healthcare resource utilization. Given these findings, *K. kingae* should be considered more frequently in the diagnostic workup for pediatric OAIs, particularly in younger children. Further research is needed to refine diagnostic methods and better understand the clinical variables, such as age and inflammatory markers, that influence pathogen-specific differences, in order to help optimize treatment strategies.

## Figures and Tables

**Table 1:** Characteristics of the study sample.

Total	702
Sex (male)—no. (%)	409 (58.3)
Age (years)—median (IQR)	4.2 (1.4, 10.4)
Diagnosis—n (%)	
Osteomyelitis	246 (35.3)
Arthritis	206 (29.6)
Concomitant osteoyelitis and arthritis	37 (5.3)
29 (4.2) Spondylodiscitis	179 (25.7)
246 (35.3) 206 (29.6) Other	37 (5.3)
Location—n (%)	
Head, spine, and thorax	95 (13.7)
Upper limb	111 (16.1)
Lower limb	485 (70.2)
Clinical and biological criteria	
Fever—n (%)	323 (46.0)
WBC (cells/mm^3^)—median (IQR)	10,730 (8030, 13,900)
CRP (mgdl)—median (IQR)	2.5 (0.8, 7.0)
VES (mm/h)—median (IQR)	32 (17, 49)
Bacteriological investigations—n (%)	
Pharyngeal swab	164 (27.3)
Blood colture	147 (24.0)
Biopsy colture	99 (15.7)
CRP on biopsy	195 (33.7)
Detected pathogen—n (%)	
*K. kingae*	174 (40.9)
*S. aureus*	155 (36.5)
*MRSA*	16 (3.8)
*S. pyogenes*	16 (3.8)
*M. tuberculosis*	6 (1.4)
*E. coli*	5 (1.2)
*S. hominis*	5 (1.2)
*H. influenzae*	4 (0.9)
*S. epidermidis*	4 (0.9)
*P. aeruginosa*	3 (0.7)
*Salmonella* spp.	3 (0.7)
*S. pneumoniae*	3 (0.7)
*C. albicans*	2 (0.5)
*E. cloacae*	2 (0.5)
*K. pneumoniae*	2 (0.5)
*S. intermedius*	2 (0.5)
Other	23 (5.4)
Undetected pathogen—n (%)	279 (39.6)
Imaging—n (%)	
X-ray	451 (69.7)
Ultrasound	276 (40.1)
CT scan	64 (9.2)
MRI	633 (91.9)
Scintigraphy	141 (20.3)
IV antibiotic therapy—n (%)	
Penicillin–clavulanate	241 (34.3)
Cephalosporin *	379 (54.0)
Glycopeptide **	147 (20.9)
Aminoglycoside	94 (13.4)
Linezolid	128 (18.2)
Quinolone ***	31 (4.4)
Meropenem	44 (6.3)
Rifampicin	38 (5.4)
Fosfomycin	26 (3.7)
Clindamycin	14 (2.0)
Piperacillin–tazobactam	1 (0.1)
IV antibiotic therapy (days)—median (IQR)	13 (3, 24)
OS antibiotic therapy—n (%)	
Amoxicillin–clavulanate	251 (35.8)
Quinolone	157 (22.4)
Linezolid	85 (12.1)
Cephalosporin	138 (19.7)
Clindamycin	64 (9.1)
Rifampicin	30 (4.3)
Glycopeptide	7 (1.0)
OS antibiotic therapy (days)—median (IQR)	19 (14, 24)
Total antibiotic therapy (days)—median (IQR)	31 (23, 42)
Length of stay (days)—median (IQR)	13 (5, 25)
Sequelae—n (%)	70 (10.2)

*: Ceftriaxone or other third-generation cephalosporins were primarily used. **: Either vancomycin or teicoplanin was used. ***: Ciprofloxacin was predominantly used in our patients, typically in older children.

**Table 2:** Comparison between pathogens and clinical characteristics.

	*K. kingae*	*S. aureus*	Other	*p*-Value
Total	174	171	80	
Sex (male)—n (%)	94 (54.0)	107 (62.6)	47 (58.8)	0.272
Age (years)—median (IQR)	1.5 (1.1, 2.1)	10.4 (6.0, 13.0)	6.8 (1.8, 11.9)	<0.001
Diagnosis—n (%)				
Osteomyelitis	49 (28.2)	80 (46.8)	26 (32.9)	<0.001
Arthritis	88 (50.6)	34 (19.9)	21 (26.6)	
Concomitant osteoyelitis and arthritis	19 (10.9)	10 (5.8)	2 (2.5)	
Spondylodiscitis	12 (6.9)	0 (0.0)	3 (3.8)	
Other	6 (3.4)	47 (27.5)	27 (34.2)	
Location—n (%)				
Head, spine, and thorax	23 (13.5)	11 (6.4)	12 (15.2)	0.034
Upper limb	36 (21.1)	24 (14.0)	13 (16.5)	
Lower limb	112 (65.5)	136 (79.5)	54 (68.4)	
Location (specific)—n (%)				
Shoulder/humerus	3 (1.8)	11 (6.4)	9 (11.4)	<0.001
Elbow/forearm	16 (9.4)	9 (5.3)	3 (3.8)	
Hand/wrist	17 (9.9)	4 (2.3)	1 (1.3)	
Chest	8 (4.7)	0 (0.0)	1 (1.3)	
Femur/knee	65 (38.0)	41 (24.0)	25 (31.6)	
Tibia/fibula	6 (3.5)	52 (30.4)	12 (15.2)	
Ankle/hindfoot	19 (11.1)	14 (8.2)	4 (5.1)	
Midfoot/forefoot	11 (6.4)	6 (3.5)	7 (8.9)	
Other	15 (8.8)	6 (3.5)	11 (13.9)	
Fever—n (%)	46 (26.4)	110 (64.3)	46 (57.5)	<0.001
WBC (cells/mm^3^)—median (IQR)	12,100 (10,100, 14,075)	9565 (7358, 12,858)	10,385 (7775, 15,425)	<0.001
CRP (mgdL)—median (IQR)	1.50 (0.7, 3.2)	6.10 (1.8, 11.5)	5 (1.0, 13.2)	<0.001
VES (mm/h)—median (IQR)	33 (18, 45)	38 (23, 59)	31 (19, 53)	0.109
Pharyngeal swab—n (%)	156 (92.9)	2 (1.7)	5 (8.2)	<0.001
Blood colture—n (%)	6 (5.2)	110 (68.8)	31 (42.5)	<0.001
Biopsy colture—n (%)	6 (4.7)	64 (38.6)	29 (37.2)	<0.001
PCR on biopsy—n (%)	100 (77.5)	31 (24.4)	22 (31.9)	<0.001
Imaging—n (%)				
X-ray	53 (36.8)	116 (71.2)	49 (64.5)	<0.001
Ultrasound	29 (17.0)	79 (46.7)	34 (43.6)	<0.001
CT scan	1 (0.6)	18 (10.7)	12 (15.2)	<0.001
MRI	155 (90.6)	164 (96.5)	67 (85.9)	0.011
Scintigraphy	4 (2.3)	36 (21.1)	14 (17.7)	<0.001
IV antibiotic therapy—n (%)				
Amoxicillin–clavulanate	89 (51.1)	66 (38.6)	34 (42.5)	0.059
Cephalosporin *	78 (44.8)	74 (43.3)	37 (46.2)	<0.001
Glycopeptide **	3 (1.7)	50 (29.2)	21 (26.2)	<0.001
Aminoglycoside	1 (0.6)	33 (19.3)	17 (21.2)	<0.001
Linezolid	1 (0.6)	57 (33.3)	14 (17.5)	<0.001
Quinolone ***	0 (0.0)	9 (5.3)	8 (10.0)	<0.001
Meropenem	1 (0.6)	13 (7.6)	15 (18.8)	<0.001
Rifampicin	0 (0.0)	23 (13.5)	2 (2.5)	<0.001
Fosfomycin	1 (0.6)	7 (4.1)	5 (6.2)	0.030
Clindamycin	1 (0.6)	7 (4.1)	5 (6.2)	0.030
Piperacillin–tazobactam	0 (0.0)	0 (0.0)	1 (1.2)	0.115
IV antibiotic therapy (days)—median (IQR)	3 (2, 3)	18 (8, 28)	17 (7, 31)	<0.001
OS antibiotic therapy—n (%)				
Amoxicillin–clavulanate	94 (54.0)	40 (23.4)	33 (41.2)	<0.001
Quinolone	2 (1.1)	53 (31.0)	20 (25.0)	<0.001
Linezolid	0 (0.0)	36 (21.1)	2 (2.5)	<0.001
Cephalosporin	72 (41.4)	9 (5.3)	7 (8.8)	<0.001
Clindamycin	1 (0.6)	36 (21.1)	5 (6.2)	<0.001
Rifampicin	1 (0.6)	15 (8.8)	4 (5.0)	0.002
Glycopeptide	0 (0.0)	5 (2.9)	0 (0.0)	0.023
OS antibiotic therapy (days)—median (IQR)	20 (17, 21)	21 (13, 28)	18 (14, 28)	0.231
Total antibiotic therapy (days)—median (IQR)	22 (21, 24)	36 (30, 47.50)	42 (30, 49)	<0.001
Length of stay (days)—median (IQR)	4 (3, 5)	19 (10, 30)	17 (8, 32)	<0.001
Sequelae—n (%)	4 (2.3)	26 (15.4)	15 (19.0)	<0.001

*: Ceftriaxone or other third-generation cephalosporins were primarily used. **: Either vancomycin or teicoplanin was used. ***: Ciprofloxacin was predominantly used in our patients, typically in older children.

## Data Availability

The corresponding author can provide data upon reasonable request.
